# Multisite Infections Caused by Carbapenem-Resistant Klebsiella Pneumoniae: Unveiling the Clinical Characteristics and Risk Factors

**DOI:** 10.3390/antibiotics14070721

**Published:** 2025-07-18

**Authors:** Jing Li, Shunjun Wu, Huanhuan Zhang, Xingxing Guo, Wanting Meng, Heng Zhao, Liqiang Song

**Affiliations:** Department of Respiratory and Critical Care Medicine, Xijing Hospital of Air Force Medical University, Xi’an 710032, China; jingli_1129@126.com (J.L.); 13310645845@163.com (S.W.); zhanghuanhuan2014@126.com (H.Z.); moonstar511@163.com (X.G.); mengwt85@163.com (W.M.); zhaoheng225@126.com (H.Z.)

**Keywords:** carbapenem resistant, klebsiella pneumoniae, multisite infections, risk factors, mortality

## Abstract

**Objectives**: There is a scarcity of studies on multisite infections (MSIs) caused by carbapenem-resistant Klebsiella pneumoniae (CRKP). The primary objectives of this research were to determine the clinical characteristics of CRKP MSI, and the risk factors of infection and mortality. **Methods**: Patients with a CRKP bloodstream infection (BSI) were enrolled retrospectively between January 2017 and December 2021 in Xijing Hospital, China. The risk factors for CRKP MSI and mortality were evaluated. The demographic data, clinical and microbiological characteristics, therapy and outcomes were analyzed. **Results**: Among 101 patients, 74.3% (75/101) had a diagnosis of CRKP MSI, while 25.7% (26/101) of CRKP non-MSI. The overall case fatality rate was 42.6% (43/101). Multivariate analysis indicated that previous surgery (OR 3.971, 95% CI 1.504–10.480, *p* = 0.005) and ICU admission (OR 3.322, 95% CI 1.252–8.816, *p* = 0.016) were independent risk factors for CRKP MSI. ICU admission (OR 4.765, 95% CI 1.192–19.054, *p* = 0.027), a Pitt bacteremia score (PBS) > 4 (OR 3.820, 95% CI 1.218–11.983, *p* = 0.022) and thrombocytopenia (OR 8.650, 95% CI 2.573–29.007, *p* < 0.001) were independent risk factors for mortality due to CRKP MSI. **Conclusions**: Our findings confirmed that CRKP MSIs were associated with poorer outcomes. To improve prognosis, early screening of individuals at the highest risk is vital.

## 1. Introduction

Over the past decade, carbapenem-resistant Klebsiella pneumoniae (CRKP) has become a major public health concern on a global scale [1]. Given the limited available treatments and high mortality, the World Health Organization categorized CRKP as a critical priority pathogen in 2017 [2]. In China, resistance rates for Klebsiella pneumoniae for carbapenem increased steadily between 2005 and 2020 (from 3.0 to 25.0% in imipenem and from 2.9 to 24.2% in meropenem) [3].

Numerous infections can be caused by CRKP, such as pneumonia, urinary tract infections, skin and soft tissue infections, and bloodstream infection (BSI) [4]. BSI is the most severe form of CRKP infections, with mortality ranging from 38.9% to 71.9% [5,6]. Multisite infections (MSIs) are common in BSI patients. An earlier study claimed that CRKP could be detected in additional samples from 65.9% of CRKP BSI patients [6]. MSIs often complicate the condition due to severe infections and inadequate antimicrobial therapies [7]. Therefore, an improved identification of patients at the highest risk is crucial to formulate effective intervention strategies to improve prognosis [8]. Many studies have focused on the risk factors for CRKP infections, but there are little data on CRKP MSIs.

Our study focused on CRKP BSI, especially in MSI patients. We described the clinical characteristics and antimicrobial susceptibility test results, as well as infection and mortality risk factors associated with CRKP MSIs.

## 2. Results

### 2.1. Clinical Characteristics and Antimicrobial Susceptibility

**Figure 1:**
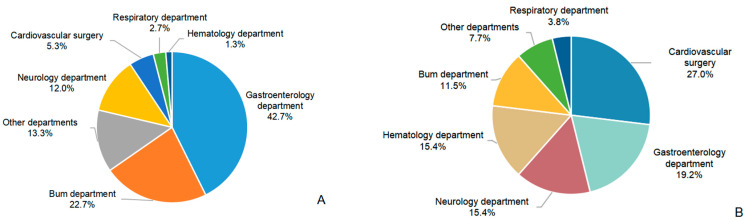
Department distribution of the MSI group (**A**) and non-MSI group (**B**). A 0.1% rounding discrepancy in (**B**) was adjusted to the largest category.

**Figure 2:**
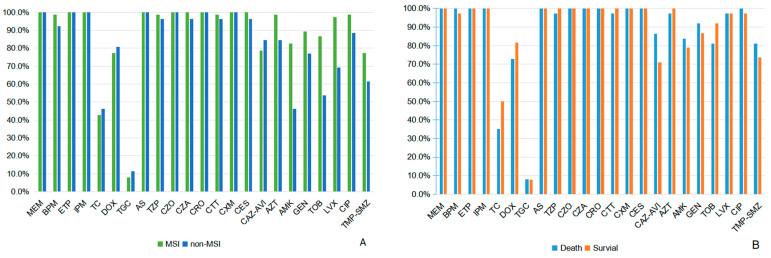
Antimicrobial resistance rate of CRKP: (**A**) MSI group and non-MSI group. (**B**) Survival group and death group in the MSI group. MEN, meropenem; BPM, biapenem; ETP, ertapenem; IPM, imipenem; TC, tetracycline; DOX, doxycycline; TGC, tigecycline; AS, ampicillin/sulbactam; TZP, piperacillin/tazobactam; CZO, cefazolin; CAZ, ceftazidime; CRO, ceftriaxone; CTT, cefotetan; CXM, cefuroxime; CES, cefoperazone/sulbactam; CAZ-AVI, ceftazidime/avibactam; AZT, aztreonam; AMK, amikacin; GEN, gentamicin; TOB, tobramycin; LVX, levofloxacin; CIP, ciprofloxacin; TMP-SMZ, co-trimoxazole. Complete resistance rates and statistical comparisons for all antimicrobials are provided in Supplementary Tables S1 and S2.

**Figure 3:**
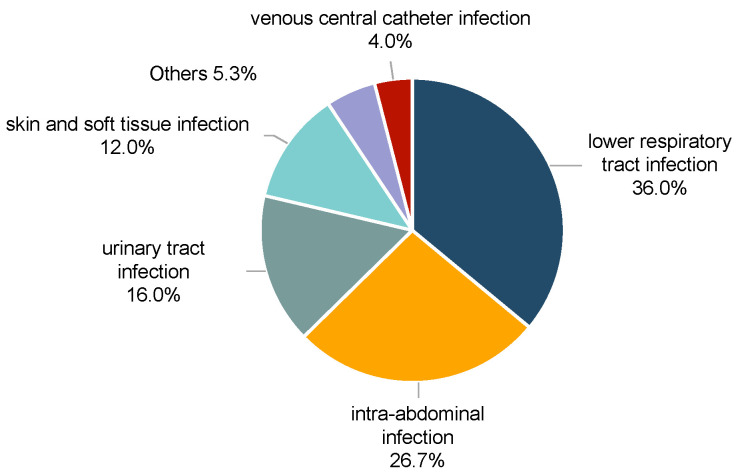
The probable infectious source of MSI.

**Figure 4:**
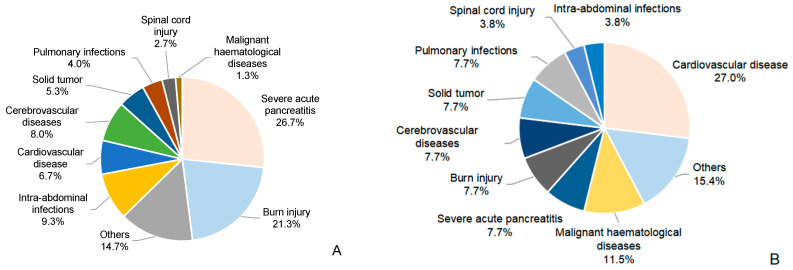
The cause of admission: (**A**) MSI group and (**B**) non-MSI group. A 0.1% rounding discrepancy in (**B**) was adjusted to the largest category.

A total of 101 patients with CRKP BSI were included, consisting of 77 male (76.2%) and 24 female (23.8%) patients, with a mean age of 52.73 ± 16.17 years. Seventy-five patients (74.3%) were classified into the MSI group, while the remaining twenty-six (25.7%) comprised the non-MSI group (Table 1). Nearly half of the CRKP MSI cases were from the gastroenterology department (42.7%), followed by the burns department (22.7%). The non-MSI group was mainly from the cardiovascular surgery and gastroenterology department (26.9% and 19.2%, respectively) (Figure 1). Drug resistance of CRKP was observed across a variety of antibiotics in both groups (Figure 2). The MSI group had significantly higher resistance rates to aztreonam (98.7% vs. 84.6%, *p* = 0.015), amikacin (81.3% vs. 46.2%, *p* = 0.001), tobramycin (86.7% vs. 53.8%, *p* < 0.001) and levofloxacin (97.3% vs. 69.2%, *p* < 0.001) (Supplementary Table S1). Tigecycline maintained the lowest resistance rates among all antibiotics tested (11.5% in MSI vs. 8.0% in non-MSI) (Supplementary Table S1). The lower respiratory tract was the most common source of infection in the MSI group (36.0%) (Figure 3). Severe acute pancreatitis was the most common cause of admission in the MSI group (26.7%) (Figure 4). The mortality rate of the MSI group was 49.3% (37/75), while that of the non-MSI group was 23.1% (6/26), which was statistically significant (*p* = 0.020).

### 2.2. Risk Factors for CRKP MSI

Besides blood samples, CRKP could be found in one site in 50.7% (38/75) patients and at least two sites in 49.3% (37/75) patients in the MSI group. Most isolates were collected from the sputum (47/75, 62.6%), followed by peritoneal effusion (28/75, 37.3%) and skin wounds (20/75, 26.7%) (Table 2). Compared with the non-MSI group, the MSI group had a higher proportion of ICU admission (73.3% vs. 46.2%, *p* = 0.011). The MSI group was more prone to receiving invasive mechanical ventilation (65.3% vs. 38.5%, *p* = 0.017). For both groups, fever was the most prevalent early clinical sign of BSI. However, chill, disturbance of consciousness, septic shock and inflammatory biomarkers did not differ between the two groups. Furthermore, 44.0% (33/75) patients received appropriate empiric therapy in the MSI group and 26.9% (7/26) in the non-MSI group (*p* = 0.125); 78.7% (59/75) patients received appropriate definitive therapy in the MSI group and 61.5% (16/26) in the non-MSI group (*p* = 0.085). Most patients in both groups received appropriate combination in definitive therapy, with 62.7% (47/75) in the MSI group and 42.3% (11/26) in the non-MSI group (*p* = 0.070).

Univariate analysis results indicated that the variables associated with CRKP MSI were as follows (Table 1): ICU admission (*p* = 0.011), invasive mechanical ventilation (*p* = 0.017), nasogastric or nasobiliary tube (*p* = 0.001), urinary catheter (*p* = 0.027), central venous catheterization (*p* = 0.010), previous surgery (*p* = 0.003) and a PBS > 4 (*p* = 0.029).

Multivariate analysis indicated that previous surgery (OR 3.971, 95% CI 1.504–10.480, *p* = 0.005) and ICU admission (OR 3.322, 95% CI 1.252–8.816, *p* = 0.016) were independent risk factors for CRKP MSIs (Table 3). The logistic regression model was credible, as indicated by a Hosmer–Lemeshow test result of *p* = 0.862.

### 2.3. Risk Factors for CRKP MSI Mortality

According to the prognosis, patients with CRKP MSIs were divided into a survival group (*n* = 38) and a death group (*n* = 37). Compared with the survival group, patients in the death group had a higher proportion of admission to the ICU (86.5% vs. 60.5%, *p* = 0.011), exposure to carbapenems (48.6% vs. 23.7%, *p* = 0.024) and received more invasive mechanical ventilation (83.8% vs. 47.4%, *p* = 0.001) (Table 4).

Multivariate analysis indicated that ICU admission (OR 4.765, 95% CI 1.192–19.054, *p* = 0.027), a PBS > 4 (OR 3.820, 95% CI 1.218–11.983, *p* = 0.022) and thrombocytopenia (OR 8.650, 95% 2.573–29.007, *p* < 0.001) were independent risk factors for mortality (Table 5). The *p*-value of the Hosmer–Lemeshow test was 0.802, indicating that the logistic regression model was valid.

## 3. Discussion

CRKP has imposed enormous threats to public health nowadays. As an important bacterial pathogen, CRKP can cause various types of nosocomial infections. Nevertheless, available treatment options for CRKP MSIs remain limited. This study primarily focused on CRKP MSIs. A high mortality up to 49.3% has been observed in patients with CRKP MSIs. Previous surgery and ICU admission were independent risk factors for CRKP MSI. ICU admission, a PBS > 4 and thrombocytopenia were associated with the mortality of CRKP MSI.

In our study, CRKP MSI was most commonly found in the gastroenterology department, and severe acute pancreatitis was the primary cause of hospitalization. Similar results were found by Yuan et al., who discovered that digestive diseases were independently associated with CRKP BSI [9]. Digestive system diseases are often accompanied by intestinal barrier function disruption and gut flora disorder [10]. Additionally, patients with severe acute pancreatitis had a more critical condition and a higher incidence of Gram-negative organism infection [11]. BSI accounts for 30–60% of the complications of severe acute pancreatitis, and most pathogens isolated from BSI patients are Gram-negative bacteria [12,13]. We, therefore, believe that patients with digestive diseases need close attention, especially those with severe acute pancreatitis.

Besides the gastroenterology department, CRKP MSI also occurred frequently in the burn department. The majority of these patients had severe burns with extensive skin barrier damage. They suffered from multiple surgeries, exposure to various antibiotics and invasive operations, all of which put them at a higher risk of contracting and spreading CRKP. Previous studies have discovered that the detection rates of CRKP in burn-injured patients were significantly higher, and these patients were more vulnerable to CRKP BSI [14,15]. Based on this information, strict contact isolation precautions are urgently needed.

It has been well established that ICU hospitalization is a risk factor for the spread of infections with multidrug-resistant bacteria. Compared with general wards, the detection rates of CRKP in the ICU were significantly higher [16,17]. In the present study, the MSI group were more likely be admitted to the ICU and had a longer length of ICU stay. The difference in the cause of admission may be primarily responsible for the result. In the MSI group, most patients suffered from severe acute pancreatitis and large-area burns. Studies have confirmed that these diseases could lead to increased susceptibility to CRKP infection [18,19]. Furthermore, patients in the ICU are critically ill and often receive various invasive treatment, such as sputum suction and endotracheal intubation. These operations can damage the mucosa, which ultimately raises the risk of MSI. The association between CRKP BSI and previous surgeries has been confirmed [6,20]. Our results also revealed that previous surgeries were an independent risk factor for CRKP MSI. Surgery was a crucial link in the treatment and substantially affected the prognosis. However, invasive procedures may damage physical barriers and result in a longer hospitalization. According to Mittal et al., hospitalization can enhance the probability of multidrug-resistant strain colonization [21]. And CRKP colonization was closely associated with nosocomial infections [22]. Impaired body barriers and infections with resistant bacteria complicate the condition, leading to a poor prognosis.

High resistance rates of CRKP to various broad-spectrum Gram-negative agents have been widely reported [4,23]. Therefore, timely adjustments of treatment based on the results of antimicrobial susceptibility test is the cornerstones of therapy. According to our findings, both the MSI group and non-MSI group were most susceptible to tigecycline. This provides a theoretical basis for us to use for treatment. However, the use of tigecycline for CRKP infections remains controversial [24]. Due to its unique pharmacokinetics, the concentration of tigecycline in tissues is much higher than in the serum. Taking standard doses of tigecycline may not reach the blood concentration that effectively kills most Gram-negative bacteria [25]. Combination therapy survival benefit for CRKP infections, though established [26,27,28], was not observed in our study. This discrepancy may stem from limited sample size, reducing the statistical power, with high baseline PBS scores masking treatment effects and restricted antibiotic options narrowing therapeutic differences. These findings warrant larger-scale validation.

In the present study, the mortality rate in the MSI group (49.3%) was significantly higher than in the non-MSI group (23.1%). Furthermore, the mortality of CRKP MSI was significantly associated with ICU admission, a PBS > 4 and thrombocytopenia, but not with gender and age. According to the study of Xu et al., CRKP patients admitted to the ICU had a higher mortality than the pooled mortality, at 48.9% and 42.14%, respectively [29]. Liu et al. and Chang et al. similarly confirmed that ICU admission increased the mortality risk of CRKP patients [30,31]. Consequently, it was not unexpected for us to find that ICU admission is a risk factor for mortality. The PBS can assess the severity of severe infections, such as BSI [32]. A higher PBS is associated with a higher mortality rate in CRKP BSI [33]. According to our findings, patients with a PBS > 4 also had a significantly higher mortality. Sezgi C et al. reported that a reduction in platelet count is an independent risk factor for patients in the ICU [34]. Moreover, the value of the platelet count in assessing condition severity and forecasting prognosis in critically ill patients was further proven by Zhang et al. [35]. Consistent with prior studies, we found that a lower platelet count was independently associated with mortality in patients with CRKP MSI. The presence of severe infection, disseminated intravascular coagulation and multiple-organ failure may be responsible for the result. No significant correlations between gender and the mortality of CRKP MSIs were found in our study, which is in line with other studies [5,6,25]. Due to poor immunity and underlying diseases, the elderly are vulnerable to developing BSI. And there was a markedly increased risk of mortality in patients aged > 65 years, indicating the complicated conditions of BSI among older adults [36]. However, in the present study, age was not found to be significantly correlated to a higher mortality for CRKP MSI. This may be related to the fact that most patients in our study were critically ill and often deteriorated rapidly to death.

Certain limitations exist in our study. First, it was a retrospective study and was conducted at a single center. Second, we did not perform the molecular characterization on CRKP isolates to determine carbapenem resistance mechanisms. Third, the definition of MSI in this study was CRKP BSI patients with other sites infection, such as urinary tract infection, pneumonia and abdominal infection. Subgroup analysis based on the number of co-infection sites is required. Lastly, while treatment strategies were analyzed, the lack of detailed pharmacological data—including specific dosages, pharmacokinetic interactions and treatment timelines—precluded deeper mechanistic analyses.

## 4. Materials and Methods

### 4.1. Design and Study Population

From January 2017 to December 2021, this retrospective study was carried out at a tertiary teaching hospital in Xi’an, China. The inclusion criterion was as follows: inpatients who met the CDC/NHSN diagnostic criteria for a CRKP BSI [37]. The exclusion criteria were as follows: (a) blood culture results indicating a polymicrobial infection and (b) missing or incomplete medical record information. After excluding specific patients, a total of 101 patients (77 males and 24 females), with an age range of 42 to 64 years (median: 52 years), were enrolled. In our analysis, only the first episode of CRKP BSI was analyzed. According to the presence of other site infections, all patients were further subdivided into the MSI group and the non-MSI group.

### 4.2. Data Collection and Definitions

The data collection included demographics, comorbidities, the cause of admission, the probable source of BSI, previous hospitalization (within one month), previous surgery (within one month), recent invasive procedures, ICU admission, length of hospital and ICU stay, antibiotic exposure and outcomes.

The detection of CRKP at the anatomical site was the prerequisite for diagnosing CRKP infection. Meanwhile, clinical signs and symptoms compatible with the infection were required. Each patient’s infection/colonization status was evaluated by two experts. CRKP BSI was defined as a positive blood culture with the presence of signs and symptoms of an infection (fever, chills or hypotension). Based on the microbiological findings and clinical symptoms, the primary site of the BSI was found. If the BSI pathogen matched the pathogen for the specific site, with a collection date in the secondary BSI attribution period, the BSI was considered secondary to the event. When the primary site could not be determined, the primary BSI was recorded. Primary sites were categorized as follows: lower respiratory tract infection, intra-abdominal infection, urinary tract infection, skin and soft tissue infection, venous central catheter infection and others.

CRKP was defined as Klebsiella pneumoniae that was not susceptible to any carbapenems (MIC ≥ 4 μg/mL for meropenem or imipenem; MIC ≥ 2 μg/mL for ertapenem) [38]. Receiving antibiotic treatment more than 48 h before a BSI was considered antibiotic exposure. The initial positive blood culture date was utilized to establish the onset of BSI. Within 24 h of a BSI being diagnosed, clinical data were collected and the Pitt bacteremia score (PBS) was calculated, with the worst value being recorded. PBS was based on five variables, including temperature, blood pressure, the need for mechanical ventilation, mental status and the presence of cardiac arrest. Single-active antibiotic treatment was defined as monotherapy, whereas multiple-active antibiotic administration was defined as combination therapy. Appropriate therapy was defined as receiving an antimicrobial with in vitro activity based on drug susceptibility results. Antimicrobial therapy administered before antibiotic susceptibility results were obtained was defined as empirical antibiotic treatment. Antimicrobial treatment based on conclusive antibiotic susceptibility results was defined as definitive antibiotic therapy.

### 4.3. Antimicrobial Susceptibility Testing

Bacterial identification was performed using the Vitek 2 system (bioMerieux, Marcy-l’Etoile, Lyon, France). The Kirby–Bauer disk diffusion method was performed to ascertain antimicrobial susceptibility to some agents: cefoperazone/sulbactam, tetracycline, cefuroxime, ceftazidime/avibactam, meropenem, biapenem, doxycycline and tigecycline. Other antibiotics susceptibility tests for the strains were examined by Gram-negative susceptibility (GNS) cards on the Vitek system (bioMérieux, Marcy-l’Etoile, France). All tests were performed according to the Clinical and Laboratory Standards Institute (CLSI) guidelines (CLSI M100). And findings were interpreted in terms of the guidelines of CLSI M100. Controls for antimicrobial susceptibility testing were Pseudomonas aeruginosa ATCC 27853 and Escherichia coli ATCC 25922.

### 4.4. Statistical Analysis

Continuous variables are expressed as the means and standard deviations (SD) if normally distributed, otherwise as the median and interquartile range (IQR). Depending on their distribution, continuous variables were compared using either the Mann–Whitney U test or Student’s *t*-test. Frequencies and proportions were used to describe categorical variables. Chi-square test or Fisher’s exact test were used to compare categorical variables. Variables with *p* < 0.05 in the univariable analysis were entered into the multivariate logistic model. SPSS version 22.0 (IBM, Armonk, NY, USA) was used for statistical analysis.

## 5. Conclusions

In summary, CRKP MSI complicated the condition and treatment, and thus eventually led to a significantly higher mortality. Previous surgery and ICU admission were independent risk factors for CRKP MSI. ICU admission, a PBS > 4 and thrombocytopenia were independent predictor of mortality, whereas other clinical factors, such as gender and age, were not correlated with mortality. Understanding these risks may contribute to identifying high-risk populations and formulating more intervention strategies to decrease the risk of developing CRKP MSI and mortality.

## Figures and Tables

**Table 1:** Comparison of clinical characteristics between the MSI group and non-MSI group.

	MSI Group *(n* = 75)	Non-MSI Group (*n* = 26)	*p*-Value ^a^
Sex			0.041
Female	14 (18.7%)	10 (38.5%)	
Male	61 (81.3%)	16 (61.5%)	
Age, years, mean ± SD	52.15 ± 16.77	54.42 ± 14.48	0.539 ^b^
Age > 65	16 (21.3%)	7 (26.9%)	0.558
ICU admission	55 (73.3%)	12 (46.2%)	0.011
Carbapenem exposure	27 (36.0%)	6 (23.1%)	0.226
Comorbidities			
Hypertension	18 (24.0%)	7 (26.9%)	0.766
Coronary heart disease	5 (6.7%)	4 (15.4%)	0.230
Diabetes mellitus	10 (13.3%)	7 (26.9%)	0.132
Chronic renal failure	14 (18.7%)	4 (15.4%)	1
Chronic liver disease	7 (9.3%)	2 (7.7%)	1
Cerebrovascular disease	7 (9.3%)	2 (7.7%)	1
Solid tumor	5 (6.7%)	3 (11.5%)	0.421
Recent invasive procedures			
Invasive mechanical ventilation	49 (65.3%)	10 (38.5%)	0.017
Nasogastric or nasobiliary tube	56 (74.7%)	10 (38.5%)	0.001
Urinary catheter	62 (82.7%)	16 (61.5%)	0.027
CVC	64 (85.3%)	16 (61.5%)	0.010
Surgery	53 (70.7%)	10 (38.5%)	0.003
CRRT	14 (18.7%)	2 (7.7%)	0.229
Events on the onset of BSI			
Fever	58 (77.3%)	21 (80.8%)	0.715
Chill	22 (29.3%)	6 (23.1%)	0.539
Septic shock	12 (16%)	3 (11.53%)	0.754
PBS > 4	29 (38.7%)	4 (15.4%)	0.029
Thrombocytopenia	28 (37.3%)	11 (42.3%)	0.653
Laboratory findings, median (IQR)			
Leukocyte count, ×10^9^/L	9.51 (5.46,14.62)	7.73 (4.32,13.87)	0.268 ^b^
Hemoglobin, g/L	95 (83,108)	97 (75.25,113)	0.636 ^b^
Albumin, g/L	31.80 (28,35.50)	31.70 (29.28,36.43)	0.522 ^b^
Appropriate empiric therapy	33 (44.0%)	7 (26.9%)	0.125
Appropriate definitive therapy	59 (78.7%)	16 (61.5%)	0.085
Combination therapy	47 (62.7%)	11 (42.3%)	0.070
Mortality	37 (49.3%)	6 (23.1%)	0.020

Data are expressed as frequencies and percentages in parenthesis, unless designated otherwise. SD, standard deviation; IQR, interquartile range; ICU, intensive care unit; CVC, central venous catheterization; CRRT, continuous renal replacement therapy; PBS, Pitt bacteremia score. Thrombocytopenia, platelet count < 100 × 10^9^/L. ^a^ Chi-square test or Fisher’s exact test; ^b^ Independent t-test or Mann–Whitney U test.

**Table 2:** The sites CRKPs were detected in addition to the blood sample.

	MSI Group (*n* = 75)	% ^a^
1 site	38	50.7 ^b^
Sputum	17	44.7
Urine	2	5.3
Peritoneal effusion	14	36.8
Skin wounds	3	7.9
Bile	1	2.6
Catheter tip	1	2.6
2 sites	29	38.7 ^b^
Sputum + Catheter tip	3	10.3
Sputum + Peritoneal effusion	8	27.6
Sputum + Urine	6	20.7
Sputum + Skin wounds	6	20.7
Peritoneal effusion + Bile	1	3.4
Peritoneal effusion + Catheter tip	1	3.4
Skin wound + Peritoneal effusion	1	3.4
Skin wound + Catheter tip	2	6.9
Skin wound + Urine	1	3.4
3 sites	7	9.3 ^b^
Sputum + Urine + Catheter tip	1	14.3
Sputum + Urine + Skin wounds	1	14.3
Sputum + Peritoneal effusion + Skin wounds	3	42.9
Sputum + Catheter tip + Skin wounds	1	14.3
Catheter tip + Urine + Skin wounds	1	14.3
4 sites	1	1.3 ^b^
Sputum + Catheter tip + Urine + Skin wounds	1	100

^a^ Percentage within subgroup; ^b^ Percentage in the total.

**Table 3:** Multivariate logistic regression analysis of the risk factors for CRKP MSIs.

	*p*-Value	OR	95%CI
Previous surgery	0.005	3.971	1.504–10.480
ICU admission	0.016	3.322	1.252–8.816

OR, odds ratio; CI, confidence interval; ICU, intensive care unit.

**Table 4:** Comparison of clinical characteristics between the survival group and the death group in the MSI group.

	Survival Group (*n* = 38)	Death Group (*n* = 37)	*p*-Value ^a^
Sex			0.517
Female	6 (18.8%)	8 (21.6%)	
Male	32 (84.2%)	29 (78.4%)	
Age, years, mean ± SD	54.63 ± 14.40	49.59 ± 18.75	0.195 ^b^
Age > 65	8 (21.1%)	8 (21.6%)	0.952
Previous hospitalization	26 (68.4%)	24 (64.9%)	0.744
ICU admission	23 (60.5%)	32 (86.5%)	0.011
Carbapenem exposure	9 (23.7%)	18 (48.6%)	0.024
Comorbidities			
Hypertension	9 (23.7%)	9 (24.3%)	0.948
Coronary heart disease	4 (10.5%)	1 (2.7%)	0.358
Diabetes mellitus	5 (13.2%)	5 (13.5%)	1
Chronic renal failure	6 (15.8%)	8 (21.6%)	0.517
Chronic liver disease	2 (5.3%)	5 (13.5%)	0.262
Cerebrovascular disease	3 (7.9%)	4 (10.8%)	0.711
Solid tumor	2 (5.3%)	3 (8.1%)	0.674
Recent invasive procedures			
Invasive mechanical ventilation	18 (47.4%)	31 (83.8%)	0.001
Nasogastric or nasobiliary tube	25 (65.8%)	31 (83.8%)	0.073
Urinary catheter	30 (78.9%)	32 (86.5%)	0.389
CVC	32 (84.2%)	32 (86.5%)	0.781
Surgery	26 (68.4%)	27 (73.0%)	0.665
CRRT	4 (10.5%)	10 (27.0%)	0.067
Events on the onset of BSI			
Fever	29 (76.3%)	29 (78.4%)	0.831
Chill	9 (23.7%)	13 (35.1%)	0.276
Septic shock	5 (13.2%)	7 (18.9%)	0.496
PBS > 4	9 (23.7%)	20 (54.1%)	0.007
Thrombocytopenia	6 (15.8%)	22 (59.5%)	<0.001
Laboratory findings, median (IQR)			
Leukocyte count, ×10^9^/L	8.52 (5.16,14.70)	9.52 (5.59,14.59)	0.141 ^b^
Hemoglobin, g/L	97.50 (87,108)	92 (81.50,107)	0.120 ^b^
Albumin, g/L	32.30 (27.75,36.63)	31.60 (28.40,34.90)	0.556 ^b^
Appropriate empiric therapy	15 (39.5%)	18 (48.6%)	0.424
Appropriate definitive therapy	27 (71.1%)	32 (86.5%)	0.103
Combination therapy	22 (57.9%)	25 (67.6%)	0.387

Data are expressed as frequencies and percentages in parenthesis, unless designated otherwise. SD, standard deviation; IQR, interquartile range; ICU, intensive care unit; CVC, central venous catheterization; CRRT, continuous renal replacement therapy; PBS, Pitt bacteremia score. Thrombocytopenia, platelet count < 100 × 10^9^/L. ^a^ Chi-square test or Fisher’s exact test; ^b^ Independent *t*-test or Mann–Whitney U test.

**Table 5:** Multivariate logistic regression analysis of the risk factors for mortality due to CRKP MSIs.

	*p* Value	OR	95%CI
ICU admission	0.027	4.765	1.192–19.054
PBS > 4	0.022	3.820	1.218–11.983
Thrombocytopenia	<0.001	8.650	2.573–29.007

OR, odds ratio; CI, confidence interval; ICU, intensive care unit. Thrombocytopenia, platelet count < 100 × 10^9^/L.

## Data Availability

The original contributions presented in the study are included in the article; further inquiries can be directed to the corresponding author.

## Supplementary Materials

The following supporting information can be downloaded at: https://www.mdpi.com/article/10.3390/antibiotics14070721/s1, Table S1: Comparison of Antimicrobial resistance rate of CRKP between MSI group and non-MSI group. Table S2: Comparison of Antimicrobial resistance rate of CRKP between survival group and death group in MSI group.

## Abbreviations

The following abbreviations are used in this manuscript:

MSI	Multisite infections
CRKP	Carbapenem-resistant Klebsiella pneumoniae
BSI	Bloodstream infection
SD	Standard deviation
IQR	Interquartile range
ICU	Intensive care unit
CVC	Central venous catheterization
CRRT	Continuous renal replacement therapy
PBS	Pitt bacteremia score
OR	Odds ratio
CI	Confidence interval
MEN	Meropenem
BPM	Biapenem
ETP	Ertapenem
IPM	Imipenem
TC	Tetracycline
DOX	Doxycycline
TGC	Tigecycline
AS	Ampicillin/sulbactam
TZP	Piperacillin/tazobactam
CZO	Cefazolin
CAZ	Ceftazidime
CRO	Ceftriaxone
CTT	Cefotetan
CXM	Cefuroxime
CES	Cefoperazone/sulbactam
CAZ-AVI	Ceftazidime/avibactam
AZT	Aztreonam
AMK	Amikacin
GEN	Gentamicin
TOB	Tobramycin
LVX	Levofloxacin
CIP	Ciprofloxacin
TMP-SMZ	Co-trimoxazole
