# Supplementary material for: Multisite Infections Caused by Carbapenem-Resistant Klebsiella Pneumoniae: Unveiling the Clinical Characteristics and Risk Factors
Source: Antibiotics (Basel). 2025 Jul 18;14(7):721. doi: 10.3390/antibiotics14070721 (PMC12291624; doi:10.3390/antibiotics14070721)
Supplement: Supplementary file 1 [file antibiotics-14-00721-s001.zip › antibiotics-3679205-supplementary.pdf]

Supplementary Table S1. Comparison of Antimicrobial resistance rate of CRKP between MSI group and non-MSI group

	**MSI group (*n*=75)**	**non-MSI group (*n*=26)**	***p*-value**
MEN	75 (100%)	26 (100%)	-
BPM	74 (98.7%)	24 (92.3%)	0.162
ETP	75 (100%)	26 (100%)	-
IPM	75 (100%)	26 (100%)	-
TC	32 (42.7%)	12 (46.2%)	0.757
DOX	58 (77.3%)	21 (80.8%)	0.715
TGC	6 (8.0%)	3 (11.5%)	0.884
AS	75 (100%)	26 (100%)	-
TZP	74 (98.7%)	25 (96.2%)	0.450
CZO	75 (100%)	26 (100%)	-
CZA	75 (100%)	25 (96.2%)	0.257
CRO	75 (100%)	26 (100%)	-
CTT	74 (98.7%)	25 (96.2%)	0.450
CXM	75 (100%)	26 (100%)	-
CES	75 (100%)	25 (96.2%)	0.257
CAZ-AVI	59 (78.7%)	22 (84.6%)	0.512
AZT	74 (98.7%)	22 (84.6%)	0.015
AMK	61 (81.3%)	12 (46.2%)	0.001
GEN	67 (29.3%)	20 (76.9%)	0.212
TOB	65 (86.7%)	14 (53.8%)	<0.001
LVX	73 (97.3%)	18 (69.2%)	<0.001
CIP	74 (98.7%)	23 (88.5%)	0.051
TMP-SMZ	58 (77.3%)	16 (61.5%)	0.117

Data are expressed as percentages in parenthesis. MEN, meropenem; BPM, biapenem; ETP, ertapenem; IPM, imipenem; TC, tetracycline; DOX, doxycycline; TGC, tigecycline; AS, ampicillin/sulbactam; TZP, piperacillin/tazobactam; CZO, cefazolin; CAZ, ceftazidime; CRO, ceftriaxone; CTT, cefotetan; CXM, cefuroxime; CES, cefoperazone/sulbactam; CAZ-AVI, ceftazidime/avibactam; AZT, aztreonam; AMK, amikacin; GEN, gentamicin; TOB, tobramycin; LVX, levofloxacin; CIP, ciprofloxacin; TMP-SMZ, co-trimoxazole.

Supplementary Table S2. Comparison of Antimicrobial resistance rate of CRKP between survival group and death group in MSI group

	**Survival group (*n*=38)**	**Death group (*n*=37)**	***p*-value**
MEN	38 (100%)	37 (100%)	-
BPM	37 (97.4%)	37 (100%)	1
ETP	38 (100%)	37 (100%)	-
IPM	38 (100%)	37 (100%)	-
TC	19 (50.0%)	13 (35.1%)	0.193
DOX	31 (81.6%)	27 (73.0%)	0.373

TGC	3 (7.9%)	3 (8.1%）	1
AS	38 (100%)	37 (100%)	-
TZP	38 (100%)	36 (97.3%)	0.493
CZO	38 (100%)	37 (100%)	-
CZA	38 (100%)	37 (100%)	-
CRO	38 (100%)	37 (100%)	-
CTT	38 (100%)	37 (100%)	-
CXM	38 (100%)	37 (100%)	-
CES	38 (100%)	37 (100%)	-
CAZ-AVI	27 (71.1%)	32 (86.5%)	0.103
AZT	38 (100%)	36 (97.3%)	0.493
AMK	30 (78.9%)	31 (83.8%)	0.591
GEN	33 (86.8%)	34 (91.9%)	0.711
TOB	35 (92.1%)	30 (81.1%)	0.287
LVX	37 (97.4%)	36 (97.3%)	1
CIP	37 (97.4%)	37 (100%)	1
TMP-SMZ	28 (73.7%)	30 (81.1%)	0.444

Data are expressed as percentages in parenthesis. MEN, meropenem; BPM, biapenem; ETP, ertapenem; IPM, imipenem; TC, tetracycline; DOX, doxycycline; TGC, tigecycline; AS, ampicillin/sulbactam; TZP, piperacillin/tazobactam; CZO, cefazolin; CAZ, ceftazidime; CRO, ceftriaxone; CTT, cefotetan; CXM, cefuroxime; CES, cefoperazone/sulbactam; CAZ-AVI, ceftazidime/avibactam; AZT, aztreonam; AMK, amikacin; GEN, gentamicin; TOB, tobramycin; LVX, levofloxacin; CIP, ciprofloxacin; TMP-SMZ, co-trimoxazole.
